# Salivary Histatin 1 and 2 Are Targeted to Mitochondria and Endoplasmic Reticulum in Human Cells

**DOI:** 10.3390/cells9040795

**Published:** 2020-03-26

**Authors:** Dandan Ma, Wei Sun, Kamran Nazmi, Enno C. I. Veerman, Floris J. Bikker, Richard T. Jaspers, Jan G. M. Bolscher, Gang Wu

**Affiliations:** 1 Department of Oral Biochemistry, Academic Centre for Dentistry Amsterdam (ACTA), University of Amsterdam (UvA) and Vrije Universiteit Amsterdam (VU), Gustav Mahlerlaan 3004, 1081 LA Amsterdam, The Netherlands; d.ma@acta.nl (D.M.); w.sun@acta.nl (W.S.); k.nazmi@acta.nl (K.N.); e.veerman@acta.nl (E.C.I.V.); f.bikker@acta.nl (F.J.B.); j.bolscher@acta.nl (J.G.M.B.); 2 Laboratory for Myology, Department of Human Movement Sciences, Faculty of Behavioral and Movement Sciences, Amsterdam Movement Sciences, Vrije Universiteit Amsterdam (VU), Van der Boechorststraat 7, 1081 BT Amsterdam, The Netherlands; r.t.jaspers@vu.nl; 3 Department of Oral Implantology and Prosthetic Dentistry, Academic Centre for Dentistry Amsterdam (ACTA), University of Amsterdam (UvA) and Vrije Universiteit Amsterdam (VU), Gustav Mahlerlaan 3004, 1081 LA Amsterdam, The Netherlands

**Keywords:** histatin, uptake, mitochondria, endoplasmic reticulum, oral saliva

## Abstract

Human salivary histatin 1 (Hst1) and Hst2 exhibit a series of cell-activating properties (e.g., promoting adhesion, spreading, migration and metabolic activity of mammalian cells). In contrast, Hst5 shows an anti-fungal property but no cell-activating properties. Previous findings suggest that their uptake and association with subcellular targets may play a determinant role in their functions. In this study, we studied the uptake dynamics and subcellular targets of Hst1, Hst2 and Hst5 in epithelial cells (HO1N1 human buccal carcinoma epithelial cell line). Confocal laser scanning microscopy (CLSM) revealed that fluorescently labeled Hst1 (F-Hst1) was taken up into the intracellular space of epithelial cells. Then, 60 min post-incubation, the total fluorescence of cell-associated F-Hst1, as measured using flow cytometry, was significantly higher compared to those of F-Hst2 and F-Hst5. In contrast, virtually no association occurred using the negative control—scrambled F-Hst1 (F-Hst^scr^). CLSM images revealed that F-Hst1, 2 and 5 co-localized with mitotracker^TM^-labeled mitochondria. In addition, F-Hst1 and F-Hst2 but neither F-Hst5 nor F-Hst1^scr^ co-localized with the ER-tracker^TM^-labeled endoplasmic reticulum. No co-localization of Hst1, 2 and 5 with lysosomes or the Golgi apparatus was observed. Furthermore, Hst1 and Hst2 but not Hst5 or Hst1^scr^ significantly promoted the metabolic activity of both human epithelial cell lines, HaCaT human keratinocytes and primary human gingival fibroblasts.

## 1. Introduction

The histatin (Hst) peptide family, which comprises at least 12 low molecular weight histidine-rich peptides, is mainly found in saliva of higher primates [1]. According to their biological functions Hsts can be divided into two major groups: (1) cell-activating Hsts (Hst1 and Hst2) [2,3,4,5,6,7] and (2) anti-fungal Hsts (e.g., Hst5) [8,9,10,11]. Hst1 and Hst2 showed a series of cell-activating properties in human and other mammalian cells, such as promotion of adhesion, spreading, migration and cell-cell junction of epithelial cells [2,5,7] on both bioactive [5] and bio-inert surfaces [7]. Comparable cell activating effects for Hst1 and Hst2 have also been found on endothelial cells, fibroblasts and osteogenic cells [5,12]. Hst1 also stimulates cell metabolic activity [13] and maintains cell viability under various adverse conditions, such as the presence of zoledronic acid [14] or ultraviolet radiation [15]. Interestingly, Hst1 and Hst2 are internalized in epithelial cells [2,7] in contrast to the D-enantiomer of Hst2 (D-Hst2), which can not promote the migration of epithelial cells and neither is internalized by the cells [3]. This suggests that activation and/or migration of Hst1 into the cell involves a stereospecific interaction with a cellular target.

In contrast to Hst1 and Hst2, Hst5 displays only weak cell-activating properties [2], but has a potent anti-fungal activity instead [2,7,16,17,18,19]. Strikingly, the anti-fungal activity of Hst5 seems dependent on its uptake by the target cell [20]. These data suggest that entry of Hsts into their target cells is a pre-requisite for their biological functions. In the present study, we mainly focused on the uptake and intracellular localization of Hst1 and Hst2 in human cells in viro with an aim to explore the potential mechanisms underlying their cell-activating effects. We adopted Hst5 as a negative control due to its weak cell-activating property. We have examined the uptake of Hst1, Hst2 and Hst5 by human cells using flow cytometry. Using confocal laser scanning microscopy (CLSM) in combination with fluorescent markers specific for cellular organelles, we have studied their subcellular targets. It was found that Hst1 and Hst2 co-localized with mitochondria as well as the endoplasmic reticulum (ER). Hst5 was also targeted to mitochondria, but not to the ER.

## 2. Materials and Methods

### 2.1. Solid-Phase Peptide Synthesis

The peptides (Table 1) were manufactured by solid-phase peptide synthesis using 9-fluorenylmethoxycarbonyl (Fmoc)-chemistry on a SyroII synthesizer (Biotage, Uppsala, Sweden), essentially as published [21] (Table 1). Peptide synthesis-grade solvents were obtained from Actu-All Chemicals (Oss, The Netherlands). The preloaded Nova-Syn TGA resins were obtained from Nova Biochem (Merck Schuchardt, Hohenbrunn, Germany) and the Fmoc amino acids were obtained from Orpegen Pharma (Heidelberg, Germany) or Iris Biotech (Marktredwitz, Germany). The fluorescently labeled Hsts (F-Hst) were manufactured on resin using the fluorescent dye ATTO-647 (A647; ATTO-TEC, Siegen, Germany). An equimolar amount of the dye was coupled to the ε-amino group of the side chain of lysine residue number 17 (lys17, K of Hst1 and the corresponding lys residues of the other Hst variants) after removal of the specific protective (ivDde)-OH group by hydrazine (2% hydrazine hydrate). Subsequently the peptides were detached from the resin and deprotected. In our previous studies, we have shown that the linkage of a large fluorescenct molecule, e.g., Fluorescein isothiocyanate (FITC) to Hst1 did not influence the stimulating effect of Hst1 on cell migration [2]. Furthermore, a biotinylated variant of Hst1 where biotin was linked to the lysine 17, displayed a comparable activity as the native peptide [21]. These results showed that the introduction of large fluorescent molecule or bulky biotin group has no adverse effects on the biological activity, exhibited similar biological and cell-binding activities as the unlabeled parent peptide.

The peptides were purified by preparative reverse phase (RP)-HPLC; Dionex Ultimate 3000 (Thermo Scientific, Breda, The Netherlands) on a Grace Spring column (250 × 25mm; Grace, Deerfield, IL, USA) containing 10 µm C18 TP beads (Vydac, Hesperia, CA, USA). Elution was performed with a linear gradient from 15 to 45% acetonitrile containing 0.1% trifluoroacetic acid (TFA) at a flow rate of 20 mL/min for 20 min. The absorbance of the column effluent was monitored at 214 nm, and peak fractions were pooled and lyophilized. Reanalysis by RP-HPLC on an analytic C18-column (218MS54; Vydac) developed with a similar gradient at a flow rate of 1 mL/min revealed a purity of at least 95%. The authenticity was confirmed by mass spectrometry with a Microflex LRF matrix-assisted laser desorption ionization time of flight mass spectrometer, equipped with an additional gridless reflectron (Bruker Daltonik GmbH, Bremen, Germany), as described previously [21].

### 2.2. Cell Culture

The human buccal epithelial carcinoma cell line HO1N1 was provided by the Japanese Collection of Research Bio-resources (Osaka, Japan). Cells were grown in Dulbecco’s modified Eagle’s medium supplemented with Nutrient Mixture F-12 (DMEM/F-12, Invitrogen, Carlsbad, CA, USA). The immortalized human keratinocyte cell line HaCaT was purchased from Cell Line Service (DKFZ, Eppel-heim, Germany). Cells were grown in Dulbecco’s modified Eagle’s medium (DMEM) supplemented with 2 mM L-glutamine. Primary fibroblasts isolated from human gingiva by dr. T.J. de Vries (ACTA) were cultured in DMEM. All media were supplemented with 10% fetal calf serum (FCS; Invitrogen, Carlsbad, CA, USA), penicillin and streptomycin (10 units/mL and 10 μg/mL, respectively; Sigma-Aldrich, St. Louis, MO, USA). Cells were cultured in 75-cm^2^ cell culture flasks at 37 °C in a 5% CO_2_ atmosphere. All the cells were maintained until near confluence, detached with 0.25% trypsin-EDTA (Invitrogen, Carlsbad, CA, USA), counted in a hepacytometer and seeded into new flasks or multiwell plates at the required cell densities.

### 2.3. Fluorescence Microscopy

To analyze the uptake of ATTO647N labeled Hst1 (F-Hst1) by HO1N1 cells, cells from a semi-confluent cell culture were transferred 10 mm μ-Dishes (Ibidi GmbH, Munich, Germany) at a density of about 1 × 10^4^ cells/well and cultivated at 37 °C for at least 24 h. Subsequently, the cells were washed once with Dulbecco’s phosphate-buffered saline (DPBS; Invitrogen, Carlsbad, CA, USA) after which serum-free medium was added. For live cell imaging, the cells were incubated with F-Hst1 at final concentrations 2μM for desired time, then washed with PBS. Then, 2 μM was used since our pilot study showed that the uptake dynamics of Hsts at 0.02, 0.1, 0.5 and 2.5 μM were similar to each other. Furthermore, in our previous study, we found that 2 μM F-Hst gave the optimal fluorescence signal for CLSM observation [6]. The cells were studied with the EVOS-FL microscope (Thermo Fisher Scientific, Waltham, MA, USA) with a 40× objective with a phase contrast setting and with a ‘Cy-5 light cube’ with a 628/40 excitation filter and a 692/40 nm emission filter. Digital photographs were recorded with a computer integrated in the microscope.

### 2.4. Flow Cytometry

HO1N1 cells were grown in 12-well cell culture plates at a starting density of about 2 × 10^5^ until semi confluence as described above. Cells were washed with DPBS and incubated in 1 mL DMEM/F12 without serum, supplemented with F-Hst variants at final concentrations of 2 μM for 10 min up to 60 min. Subsequently the cells were washed with DPBS and harvested by incubation with 0.05% trypsin-EDTA for 4 min, followed by gentle aspiration of the trypsin-EDTA (carefully to not remove the cells), and cells were detached by gently pipetting with 1 mL DMEM/F12 with serum to block the remnants of trypsin activity. Subsequently the cell suspensions were transferred to 2 mL vials and centrifuged at 4 °C, 500 rpm in an Eppendorf 5810R centrifuge (Eppendorf, Hamburg, Germany) for 10 min. The supernatant was removed and the pellet was resuspended in 200 µL of DPBS containing Ca^2+^/Mg^2+^ and supplemented with 0.1% FCS.

All samples were kept on ice till analyzed at room temperature using a BD Accuri^®^ C6 Plus Flow Cytometer (BD Biosciences, Piscataway, NJ, USA). A minimum of 10,000 events were acquired using the following settings: flowrate fast (66 µL/min), and forward scatter (FSC) threshold of 500.000. Based on the scatterplots the viable cells were gated and the intensity of cell-associated fluorescence was determined in the FL-4 channel. Data were analyzed using BD AccuriCFlow^®^ Software (BD Biosciences) and Microsoft Excel. Arbitrary fluorescence unit (AFU) was recorded to indicate the fluorescence intensity. All experiments were performed in triplicates.

### 2.5. Cellular Internalization of the F-Hsts

To study the internalization of the F-Hst and variants by HO1N1 cells 1 × 10^4^ cells were distributed into 10 mm μ-Dishes (Ibidi GmbH, Munich, Germany) growth medium. After 24 h, the cells were washed with DPBS for 3 times. Thereafter, cells were incubated with NucBlue™ live cell stain following the manufacturer’s protocol (Invitrogen, Carlsbad, CA, USA). Cell membrane was labeled using PKH67GL (Sigma-Aldrich, St. Louis, MO, USA), following manufacturer’s instructions. Then serum-free DMEM/F12 containing the F-Hst variants at a final concentration of 2 μM. After 60 min of incubation with F-Hst variants, the cells were washed with DPBS. Subsequently, the cells were studied by a confocal laser scanning microscopy (CLSM) system (TCS SP8, Leica, Wetzlar, Germany) equipped with a 63 × 1.4 NA oil objective, and we chose the No. 20 Z-slice from the bottom (approximately 50 Z-slices per cell) since this slide contained the largest cross-section of nuclei.

### 2.6. Subcellular Localization of the F-Hsts

Prior to imaging, nuclear DNA was stained with NucBlue™ live cell stain following the manufacturer’s protocol (Invitrogen, Carlsbad, CA, USA). Cells were stained with CytoPainter lysosomal staining kit (Abcam, Cambridge, MA, USA), Golgi apparatus staining kit (Abcam, Cambridge, MA, USA) following the manufacturer’s protocol. For detection of mitochondrial and ER, cells were washed with DPBS and incubated with 500 nM MitoTracker^®^ Green FM (Invitrogen, Carlsbad, CA, USA) and 500 nM ER-Tracker Blue-White DPX (Invitrogen, Carlsbad, CA, USA) for 30 min following the manufacturer’s protocol. Staining solution was replaced with cell culture media and cells were immediately imaged. Confocal stacks were achieved with a Leica TCS SP8 using a 63 × 1.4 NA oil objective. The confocal images were taken after a 60 min incubation with various Hsts. Co-localization was quantified using Coloc2 in Fiji. The Pearson’s correlation coefficient (P coloc value), describes the relationship in intensity distribution between two color channels, and the value ranges from −1 to 1. Values between −1 and 0.5 suggest no co-localization, P coloc value > 0.5 suggests some degree of co-localization and P coloc value = 1 suggests complete co-localization.

### 2.7. Cellular Metabolic Activity Assay

We evaluated the influence of the histatins and variants on cellular metabolic activity with the resazurin-based PrestoBlue reagent according to the manufacturer’s instructions (Invitrogen, Carlsbad, CA, USA). HO1N1, HaCaT and gingival fibroblasts were seeded into 96-well plates at a density of 1 × 10^4^ per well, in 90 μL of cell culture medium, and incubated for 24 h to allow cell adherence. Cells were then incubated in the presence or absence of Hst1 and their variants for 24 h. Untreated cells were used as a negative control and cells treated with 10 ng/mL recombinant human epithelial growth factor (rhEGF) as positive control as previously described [4]. The PrestoBlue solution (10 μL) was added into each well after 30 min, after which absorbance was measured at excitation wavelength 570 nm and emission wavelength 600 nm. Data were presented as folds of optical density (OD) of absorbance by normalizing the OD value in other groups normalized to that of the respective control group.

### 2.8. Statistical Analysis

All quantitative data in this study represent the mean value ± standard deviation (SD) for n ≥ 3 (number of experiments). Significance levels were determined by one-way analysis of variance (ANOVA) unless explicitly stated otherwise (GraphPad Prism 7).

## 3. Results

### 3.1. Uptake Dynamics of the F-Hsts

**Figure 1:**
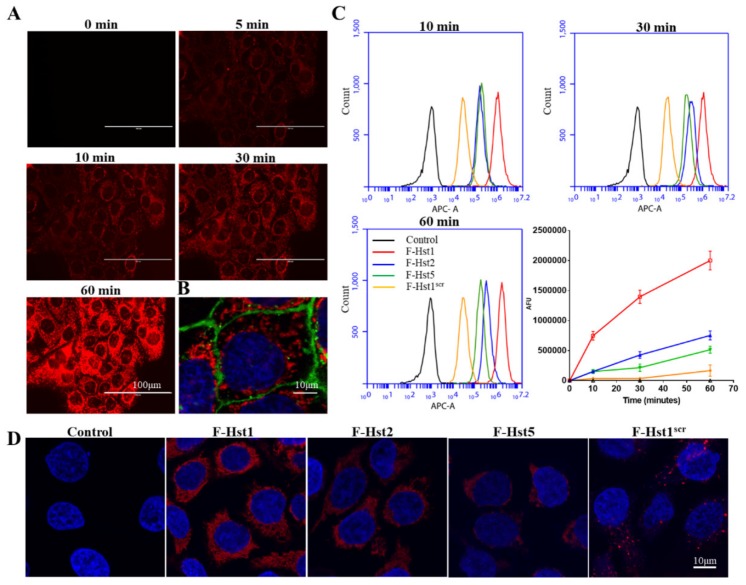
Cellular uptake of the ATTO-647 labeled Hst1 (F-Hst1), F-Hst2, F-Hst5 and F-Hst1^scr^ by human buccal epithelial carcinoma HO1N1 epithelial cells. (**A**) Fluorescent micrographs showed that the association of F-Hst1 (in red) with the cells became detectable as early as 5 min and increased with time; (**B**) confocal laser scanning microscopy (CLSM) image showed F-Hst1 (in red) distributed mainly between cell membrane (in green) and nuclei (in blue), which indicated that F-Hst1 was internalized into intracellular space; (**C**) flow cytometric analysis showed the time-course fluorescence density of cell-associated F-Hst1, F-Hst2, F-Hst5 and F-Hst1^scr^. At all the time points (10, 30 and 60 min), F-Hst1 showed significantly higher fluorescence density than F-Hst2 or F-Hst5. In contrast, Hst^scr^ showed only mild fluorescence density. Data were presented as mean ± SD. (**D**) In line with the flow cytometric data, CLSM images showed that the highest level of accumulation in epithelial cells was observed with F-Hst1, followed by F-Hst2 > F-Hst5 > F-Hst1^scr^.

Fluorescence microscopy revealed that a detectable association of F-Hst1 (in red) with HO1N1 epithelial cells was found as early as 5 min after starting the incubation (Figure 1A). The association of F-Hst1 increased over the monitoring time span (60 min). CLSM image indicated the F-Hst1 mainly distributed between nuclei (in blue) and cell membrane (in green) (Figure 1B), which indicated the uptake of F-Hst1 into the intracellular space. Flow cytometry analyses were adopted to quantify the time-dependent uptake dynamics of F-Hst1, F-Hst2, F-Hst5 and F-Hst1^scr^ in epithelial cells. The control cells (without F-Hsts) showed a mean fluorescence density of about 10^2.8^ AFU (Figure 1C). After 10 min incubation, F-Hst1 uptake (with a fluorescence density of approximately 7.47 ± 0.72 × 10^5^ AFU) was significantly higher compared to F-Hst2 (1.52 ± 0.23 × 10^5^ AFU) (*p* = 0.0002) and F-Hst5 uptake (1.51 ± 0.34 × 10^5^ AFU) (*p* = 0.0002). F-Hst1^scr^ showed the lowest fluorescent counts (3.50 ± 0.21 × 10^4^ AFU). For all the F-Hsts, the cell-associated fluorescence increased between 10 min and 60 min with different rates. 60 min post-incubation, the total uptake amount of cell-associated F-Hst1 (2.00 ± 0.15 × 10^6^ AFU) was significantly higher than those of F-Hst2, F-Hst5 and F-Hst1^scr^ (0.75 ± 0.07 × 10^6^ AFU, 0.51 ± 0.06 × 10^6^ AFU and 0.17 ± 0.10 × 10^6^ AFU, respectively) (*p* = 0.0002, *p* = 0.0001 and *p* < 0.0001, respectively) (Figure 1C). Thereafter, CLSM was adopted to visualize the intracellular accumulation of the F-Hsts (Figure 1D). In line with the FACS data, the highest level of accumulation in epithelial cells was observed with F-Hst1, followed by F-Hst2 > F-Hst5 > F-Hst1^scr^ (Figure 1D).

### 3.2. Subcellular Localization of the F-Hsts 

**Figure 2:**
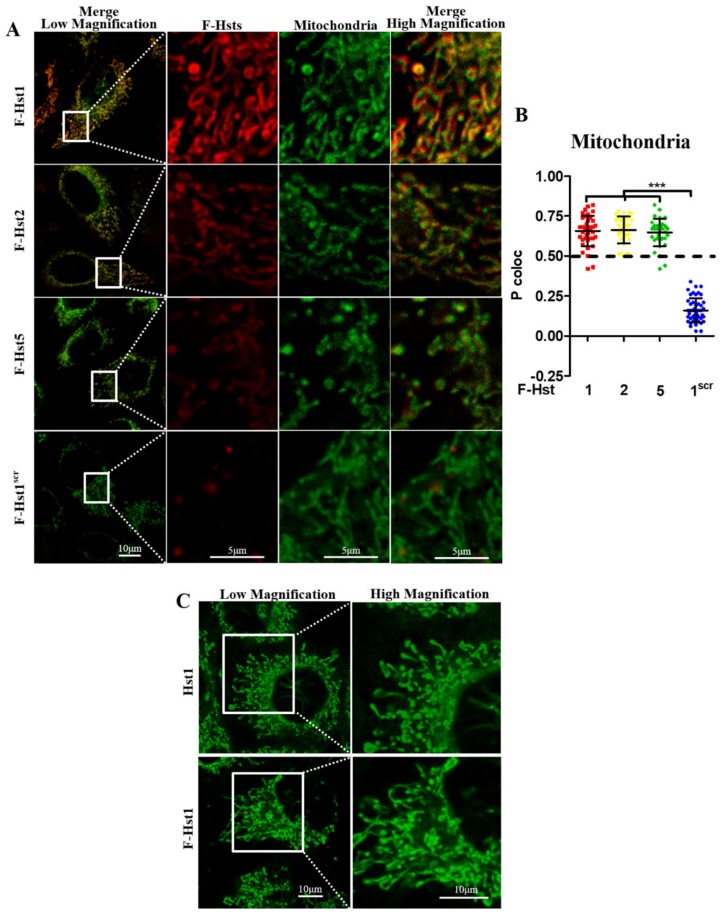
F-Hst1, F-Hst2 and F-Hst5 were targeted to mitochondria. (**A**) CLSM images showed F-Hst1, F-Hst2 and F-Hst5 but not F-Hst^scr^ (in red) showed co-localization with mitochondria (in green). (**B**) The Pearson’s correlation analysis showed that F-Hst1, F-Hst2 and F-Hst5 co-localized with mitochondria with a P coloc value of about 0.65, while F-Hst^scr^ did not show significant co-localization with mitochondria. Data were presented as mean ± SD. ***: *p* < 0.001. (**C**) CLSM images showed that F-Hst1 was associated with a similar morphological pattern of mitochondria (in green) as that of Hst1 (non-fluorescently labeled).

**Figure 3:**
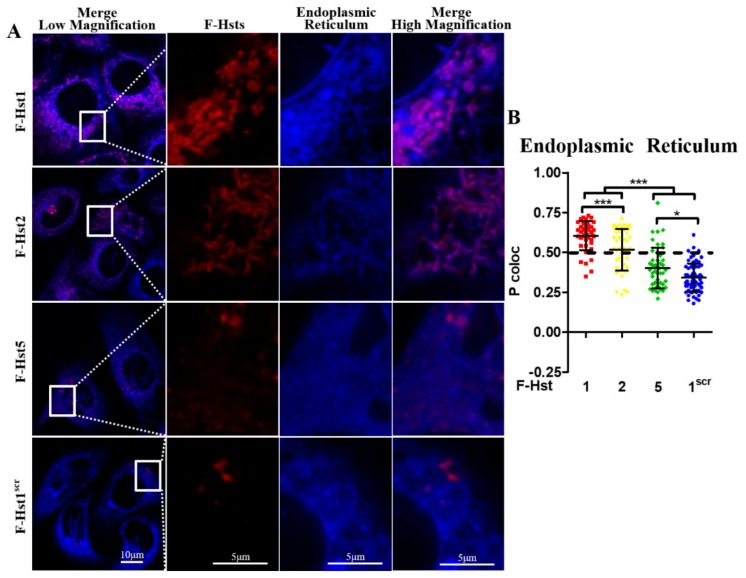
F-Hst1, F-Hst2 and F-Hst5 were targeted to endoplasmic reticulum (ER). (**A**) CLSM images showed F-Hst1, F-Hst2 and but not F-Hst5 or F-Hst^scr^ (in red) showed co-localization with ER (in blue). (**B**) The Pearson’s correlation analysis showed that F-Hst1 and F-Hst2 co-localized with ER (with P coloc values of 0.60 and 0.51, respectively), while but not F-Hst^scr^ or F-Hst5 did not show significant co-localization with mitochondria. Data were presented as mean ± SD. *: *p* < 0.05; ***: *p* < 0.001.

**Figure 4:**
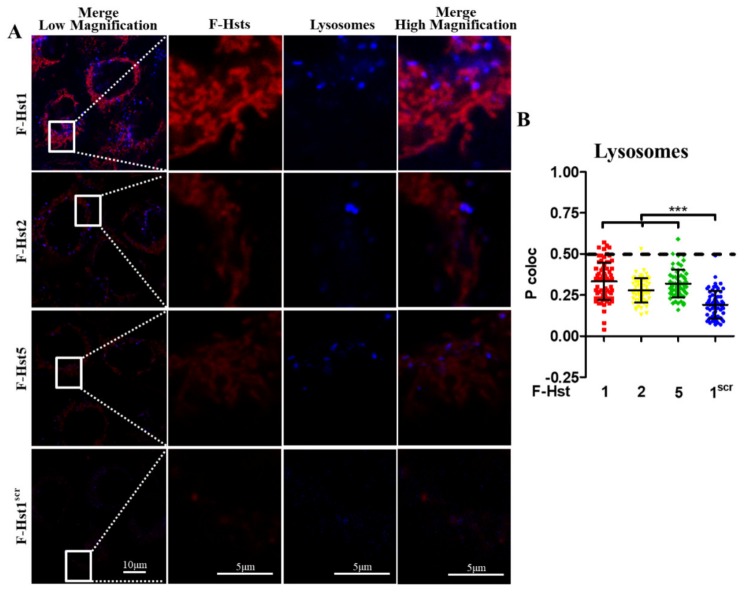
F-Hst1, F-Hst2 and F-Hst5 were not targeted to lysosomes. (**A**) CLSM images showed neither F-Hst1, F-Hst2 F-Hst5 nor F-Hst^scr^ (in red) showed a co-localization with lysosomes (in blue). (**B**) The Pearson’s correlation analysis also confirmed that none of F-Hst1, F-Hst2 F-Hst5 or F-Hst^scr^ co-localized with lysosomes (P coloc value below 0.5). Data were presented as mean ± SD. ***: *p* < 0.001.

**Figure 5:**
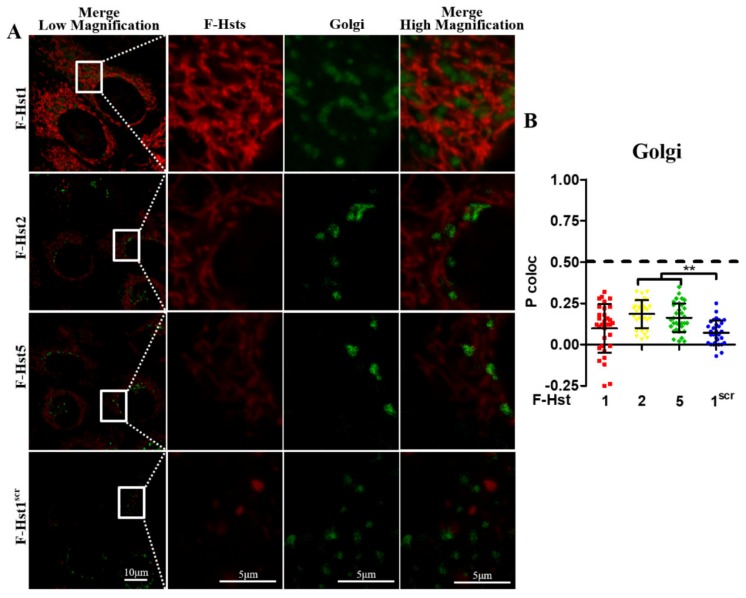
F-Hst1, F-Hst2 and F-Hst5 were not targeted to Golgi. (**A**) CLSM images showed neither F-Hst1, F-Hst2 F-Hst5 nor F-Hst^scr^ (in red) showed a co-localization with Golgi (in green). (**B**) The Pearson’s correlation analysis also confirmed that none of F-Hst1, F-Hst2 F-Hst5 or F-Hst^scr^ co-localized with lysosomes (P coloc value below 0.5). Data were presented as mean ± SD. **: *p* < 0.01.

**Figure 6:**
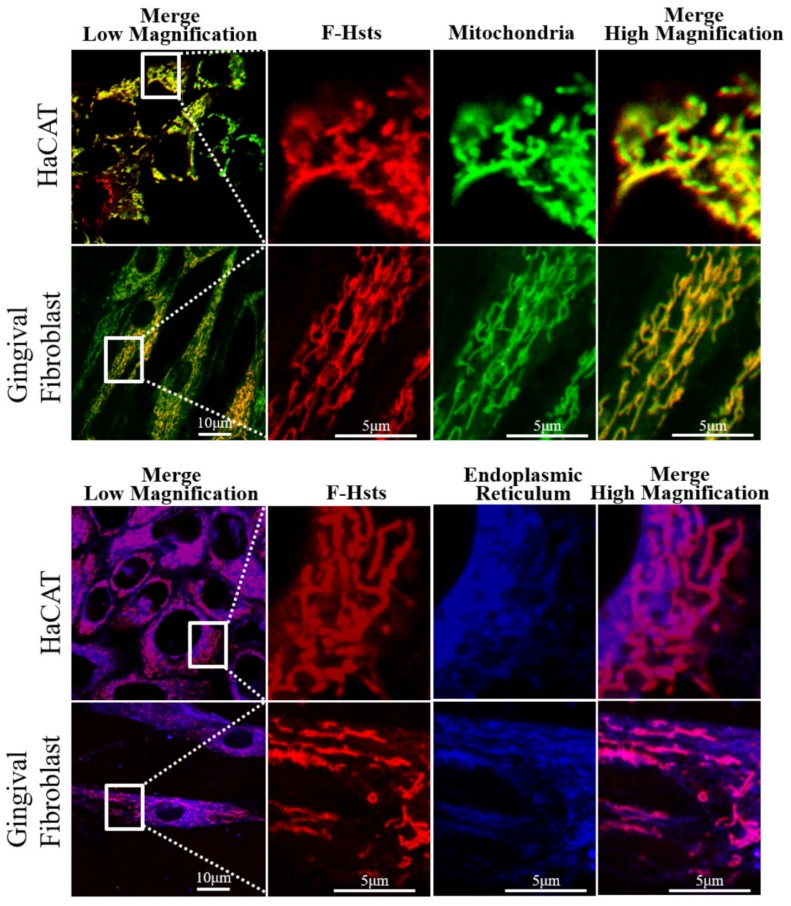
CLSM images showed that F-Hst1 (in red) was also targeted to mitochondria (in green) and endoplasmic reticulum (ER) (in blue) in both HaCaT epithelial cell line and primary human gingival fibroblasts as in HO1N1 epithelial cells.

Using cell organelle markers, the subcellular location of the various Hsts was studied in more detail (Figure 2, Figure 3, Figure 4 and Figure 5). F-Hst1 was associated with a similar morphological pattern of mitochondria (in green) as that of non-labeled Hst1 (non-fluorescently labeled) (Figure 2C). F-Hst1, F-Hst2 and F-Hst5 co-localized with mitochondria with a P coloc value of about 0.65. F-Hst1 showed a significantly (*p* = 0.0004) higher co-localization (P coloc value of 0.60 ± 0.09) with ER than F-Hst2 (P coloc value of 0.51 ± 0.13) (Figure 2B and Figure 3B). In contrast, the P coloc values of F-Hst5 and F-Hst1^scr^ with the ER (0.40 ± 0.13 and 0.36 ± 0.09) were lower than 0.5, suggesting no co-localization of F-Hst5 and F-Hst1^scr^ with the ER (Figure 3B). None of the tested Hsts showed a co-localization with Golgi apparatus (Figure 4) and lysosomes (Figure 5). Furthermore, our results show that F-Hst1 (in red) was targeted to mitochondrial (green) and ER (blue) in both HaCaT human keratinocyte cell line and primary human gingival fibroblasts (Figure 6).

### 3.3. Uptake Dynamics and Subcellular Target of Truncated F-Hsts, F-Hst1_1-11_, F-Hst1_12-22_ and F-Hst1_23-38_

**Figure 7:**
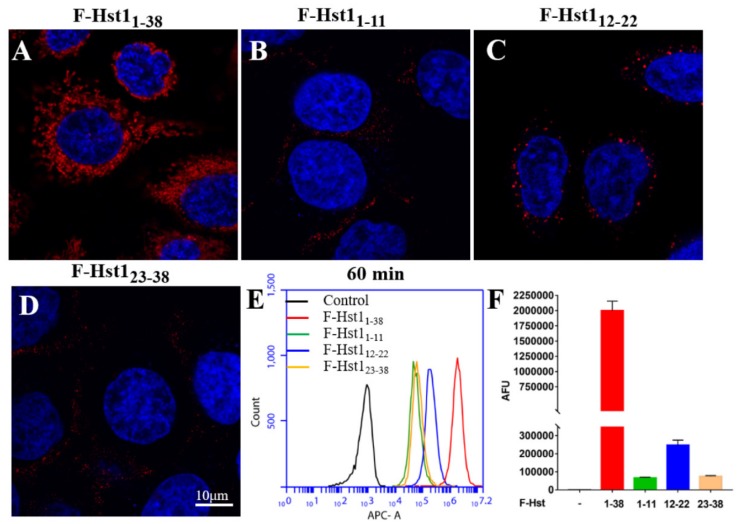
Cellular uptake of the F-Hst1 and truncated variants, such as F-Hst1_1-11_, F-Hst1_12-22_ and F-Hst1_23-38_ by HO1N1 epithelial cells. (**A**–**D**) CLSM images showed that a significantly higher amount of F-Hst1 accumulated in the vicinity of nuclei than F-Hst1_12-22_, while F-Hst1_1-11_ and F-Hst1_23-38_ showed only mild accumulation. (**E**,**F**) Flow cytometric analysis confirmed that F-Hst1 showed the highest cell-associated fluorescence density, which was followed by F-Hst1_12-22_. In contrast, F-Hst1_1-11_ and F-Hst1_23-38_ showed only mild fluorescence density. Data were presented as mean ± SD.

**Figure 8:**
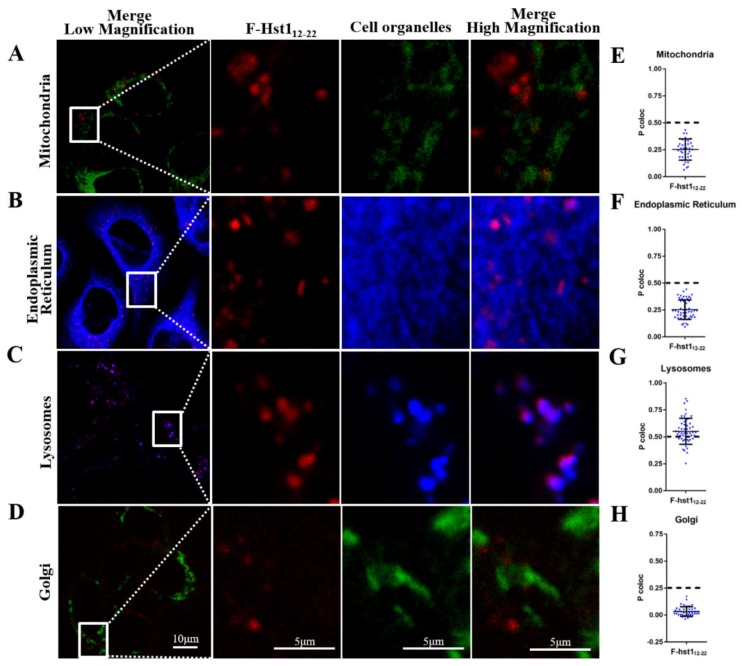
F-Hst1_12-22_ was not targeted to mitochondria and endoplasmic reticulum (ER) in HO1N1 epithelial cells. (**A**–**D**) CLSM images showed that F-Hst1_12-22_ showed co-localization with lysosomes (in blue) but not with mitochondria (in green) and ER (in blue) or Golgi (in green). (**E**–**H**) The Pearson’s correlation analysis showed that F-Hst1_12-22_ co-localize with lysosomes with a P coloc value of about 0.55, but not with mitochondria, ER or Golgi (with P coloc values below 0.5). Data were presented as mean ± SD.

From the above described results it may be deducted that the absence of amino sequences 1-11 in Hst2 and 23-38 in Hst5 (compared to Hst1) dramatically compromised their uptake rates. Consequently, we assessed the uptake dynamics and subcellular targets of truncated variants, F-Hst1_1-11_, F-Hst1_12-22_ and F-Hst1_23-38_ to explore their role in the cellular uptake of Hst1, Hst2 and Hst5. CLSM images revealed that the uptake of the truncated variants F-Hst1_1-11_, F-Hst1_12-22_ and F-Hst1_23-38_ was much lower than that of the whole molecule F-Hst1 (Figure 7A–D). Of the truncated variants, some intracellular labelling was only observed with F-Hst12-22. Uptake of F-Hst11-11 and F-Hst123-38 was comparable to that of the negative control (F-Hst1^scr^). At 60 min post-incubation Hst1 showed a mean fluorescence of 2.00 ± 0.15 × 10^6^ AFU, which was significantly higher than that of F-Hst1_12-22_ (0.25 ± 0.03 × 10^6^ AFU), F-Hst1_1-11_ (0.07 ± 0.00 × 10^6^ AFU) and F-Hst1_23-38_ (0.07 ± 0.00 × 10^6^ AFU) (Figure 7F). F-Hst1_12-22_ mainly co-localized with the lysosomal marker (P coloc value of 0.55 ± 0.12, not with markers for the mitochondria or the ER or Golgi (Figure 8).

### 3.4. Cellular Metabolic Activity

**Figure 9:**
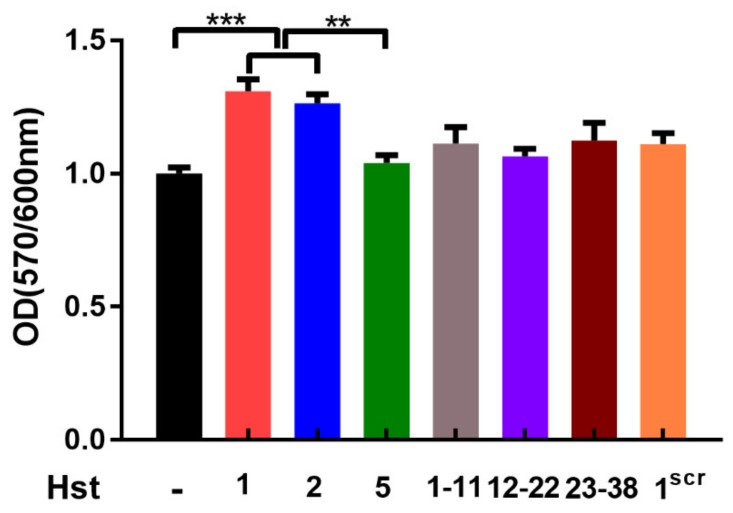
Graph depicting the effects of Hst1, Hst2, Hst5, F-Hst1_1-11_, F-Hst1_12-22_ and F-Hst1^scr^ on the cellular metabolic activity of HO1N1 human epithelial cells measured using PrestoBlue assay. The results showed that Hst1 and Hst2, but not other Hst1 variants, significantly enhanced cellular metabolic activity of HO1N1 epithelial cells in comparison with the control group (no Hsts). Data were presented as mean ± SD. **: *p* < 0.01, ***: *p* < 0.0001.

**Figure 10:**
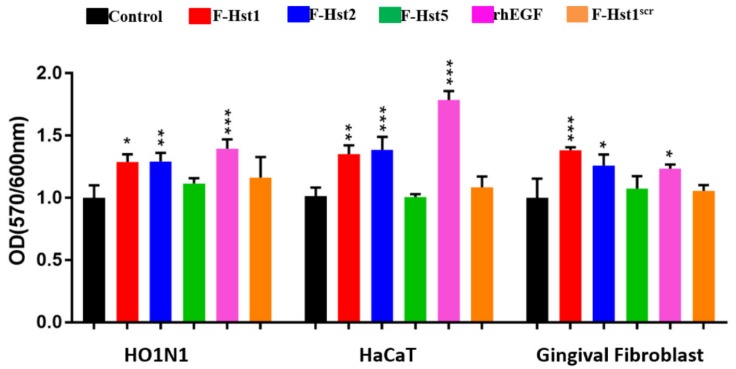
Graph depicting the effects of Hst1, Hst2, Hst5, recombinant human epithelial growth factor (rhEGF) (as a positive control) and F-Hst1^scr^ (as a negative control) on the cellular metabolic activity of HO1N1 human epithelial cells, HaCaT human keratinocytes and primary gingival fibroblasts measured using PrestoBlue assay. The results showed that Hst1 and Hst2 but not Hst5 or F-Hst1^scr^ significantly enhanced cellular metabolic activity of HO1N1 epithelial cells. The positive control rhEGF could significantly enhance the metabolic activity of all the three types of cells. Data were presented as mean ± SD. *: *p* < 0.05; **: *p* < 0.01; ***: *p* < 0.001.

We next investigated the effects of Hst variants on the metabolic activity of human HO1N1 epithelial cells. Hst1 and Hst2 significantly enhanced the cellular metabolic activity, while Hst5 or Hst^src^ or truncated Hsts (Hst1_1-11_, Hst1_12-22_ and Hst1_23-38_) did not (Figure 9). Thereafter, we investigated whether Hst1 and Hst2 could also enhance the cellular metabolic activity of HaCaT keratinocytes and primary gingival fibroblasts. Results showed that a significantly higher cellular metabolic activity was found when treated by rhEGF in comparison with the control group. Furthermore, Hst1 and Hst2 also significantly enhanced the cellular metabolic activity in HaCaT keratinocytes and primary gingival fibroblasts as they did in human HO1N1 epithelial cells. In contrast, Hst5 or Hst^src^ did not significantly influence the cellular metabolic activity of HaCaT keratinocytes and primary gingival fibroblasts either (Figure 10).

## 4. Discussion

Previous studies have suggested that the uptake and subcellular targets were critical for the biological functions of both cell-activating Hst1, Hst2 [2,7] and anti-fungal Hst5 [10,22,23]. In this study, we analyzed the uptake dynamics and subcellular targets of the Hst variants in more detail. It was previously shown that within 30 min Hst1 could be observed inside epithelial cells [7]. In this study, we found that already 5 min after addition of F-Hst1, F-Hst2 and F-Hst5 to epithelial cells, intracellular accumulation was observed. Previous studies suggest that the uptake of Hst1 and Hst2 in epithelial cells involves a stereospecific membrane receptor-mediated [2,3] and energy-dependent [2] process. Moreover, in another study was shown that in endothelial cells endosomes were involved in the functions of Hst1 [12]. However, the molecular mechanisms mediating their uptake remain largely unknown. Although all the three F-Hst variants showed a prompt uptake, at each time point the uptake of F-Hst1 was significantly higher than those of F-Hst2 and F-Hst5. This is striking, since previously it was found that equimolar concentrations of Hst1 and Hst2 exert comparable cell-activating properties to epithelial cells [2]. Furthermore, while Hst5 did not show cell-activating effects, here we found that it internalized at a comparable rate to that of Hst2. These results suggested that the interaction with a putative membrane transporter was not a direct trigger for Hst-induced cell migration. In this study, it was demonstrated that Hst1, Hst2 and Hst5 were localized in close vicinity of the mitochondria of epithelial cells. Hst5 has previously been shown to accumulate in mitochondria of *Candida albicans* and Leishmania [10,24], putatively resulting in a bioenergetic collapse and subsequent death of the target organism. It should be noted, however, that whereas Hst5 also in the epithelial cell targets to mitochondria, it does not exert cytotoxic effects [25].

In this study, we focused on the uptake and localization of Hst1 by human cells in physiological conditions. Consequently, we adopted a series of staining kits for live cells, such as NucBlue™, MitoTracker^TM^ and ER-Tracker Blue-White DPX to stain nuclei, mitochondria and ER, respectively. Furthermore, we also used F-Hst1 to monitor the intracellular distribution of Hst1 in live cells. Another option would be the staining with antibodies in fixed cells, which in general gives a better resolution in comparison to live cell staining. However, we found that F-Hst1 dispersed, diffused and lost its subcellular targeting property immediately after fixation with 4% paraformaldehyde in PBS. This phenomenon made it really difficult to observe the subcellular targets of Hst1 in fixed cells. In future, further tests can be performed to find a more suitable fixation method.

In our previous studies, we have already shown that linkage of of large fluorescent molecule or bulky biotin group to Hst1 did not lead to significant adverse effects on its biological activity [2,21]. Albeit so, concerns may still be raised whether the fluorescent labeling will influence the subcellular targeting property of Hst1. Therefore, we compared the distribution pattern of mitochondria in cells treated with either Hst1 (non-fluorescently labeled) on the one hand and F-Hst1 on the other hand. We found that F-Hst1 was associated with a comparable morphological pattern of mitochondria (in green) to that of Hst1 (non-fluorescently labeled) (Figure 2C). This finding suggested that fluorescent labeling did not change the morphology of mitochondria or bring additional subcellular targeting property to Hst1.

**Figure 11:**
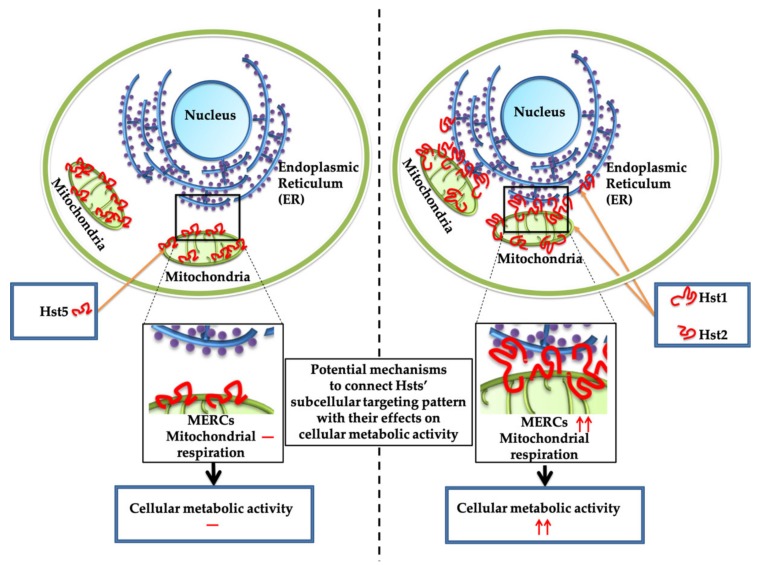
A schematic representation of cell-activating Hst1 and Hst2, which have a different subcellular targeting pattern compared to non-cell-activating Hst5. Hst1 and Hst2 co-localize with both mitochondria and ER, while Hst5 co-localizes with mitochondria but not ER. Such a subcellular targeting pattern of Hst1 and Hst2 may potentially enhance mitochondria-ER contacts (MERCs), thereby promoting mitochondria respiration and cellular metabolic activity. In contrast, Hst5 cannot regulate MERCs, thus showing no effect on cellular metabolic activity.

Interestingly, Hst1 and Hst2, but not Hst5, co-localized with the ER. It remains unveiled how the co-localization with ER might regulate the functions of Hsts. One possibility is that Hst1 and Hst2 promote the mitochondria-ER contacts (MERCs) (Figure 11), which regulates the calcium homeostasis of the cell [26]. Interestingly, oscillations in cytosolic calcium activate cytoskeletal remodeling, actin contraction and turnover processes of focal adhesion (FA), which are necessary for cell migration [27,28,29]. Secondly, mitochondria-ER coupling promotes mitochondrial respiration and bioenergetics [29,30], which may explain the stimulation of cell metabolic activity by Hst1 (Figure 11). This result is consistent with the finding in a previous literature [13]. Furthermore, cell spreading and migration are energy-demanding activities [17]. During motility, cells transiently form protruding filopodia and lamellipodia in multiple dimensions, for which ATP-dependent actin polymerization and myosin ATPase activity are the principal driving forces [31,32]. Further studies need to be performed to investigate the involvement of mitochondria-ER interaction in the cell-activating properties of Hst1 and Hst2.

In our previous publication [3], the fluorescence density of propidium iodide was measured to reflect the cell numbers so as to compare the effects of Hst1 at different concentrations on cell proliferation. In that study, there was no significant difference in cell numbers among different concentrations of Hst1. This was consistent with another study where Hst1 did not promote cell proliferation with a MTS kit as the tool to reflect cell numbers [12]. In the present study, we adopted the Prestoblue^®^ assay; when added to live cells, the PrestoBlue^®^ reagent is modified by the reducing environment of viable cells and turns red in color, becoming highly fluorescent for measurement. Consequently, the Presoblue^®^ assay is to measure the total metabolic activity of the live cells per well, which can reflect both cell number and metabolic activity. In this study, we did find the increase of the total metabolic activity. Such an increase can be largely attributed to the increase of cellular metabolic activity but not to the increase of cell numbers/cell proliferation. This is also consistent with the finding that cell-activating Hst1 and Hst2 co-localized with both ER and mitochondria. In such a way, Hst1 may promote mitochondria-ER interaction, thereby enhancing metabolic activity.

Our results indicated that the amino sequences of Hsts are highly critical for their uptake into cells and subsequent targeting to cell organelles. When compared to F-Hst1, the lack of Hst1_1-11_ in F-Hst2 and the lack of Hst1_23-38_ in F-Hst5 seemed to significantly compromise their uptake efficiencies. All the three Hsts, that share the amino sequences of Hst1_23-38_, showed a high co-localization with mitochondria. The lack of Hst1_23-38_ resulted in the absence of co-localization of F-Hst5 with ER. These results suggested that the amino acid sequences of Hst1_1-11_ and Hst1_23-38_ seemed to play an important role for uptake dynamics of F-Hsts. Furthermore, the amino acid sequence of Hst1_12-22_ might be critical for the mitochondria-targeting property, whereas that of Hst1_23-38_ may tentatively be important for targeting to the ER. Consequently, in the present study we also synthesized truncated F-Hsts, such as F-Hst1_1-11_, F-Hst1_12-22_ and F-Hst1_23-38_ and tested their uptake dynamics and subcellular targets. Interestingly, none of the truncated F-Hsts could reach the uptake level of F-Hst1, F-Hst2 and F-Hst5, which suggested that their combinations were more important than either of the separate sequences. Among the three truncated F-Hsts, F-Hst1_12-22_ was associated with the highest total uptake amount. Therefore, we analyzed the subcellular targets of F-Hst1_12-22_. F-Hst1_12-22_ did not resemble the property of F-Hst1, showing co-localization with neither mitochondria nor ER. Instead, F-Hst1_12-22_ showed a high co-localization with lysosomes. Consequently, none of the truncated F-Hsts alone could mimic the uptake and subcellular targeting properties of F-Hst1, F-Hst2 or F-Hst5. These findings indicated that mitochondria-targeting property could only be realized when Hst1_12-22_ linked to Hst1_1-11_ or Hst1_23-38_. Hitherto, it remains unclear how Hsts are internalized and targeted to mitochondria and ER. A previous study suggested that early endosomes were involved in the cell-activating effect of Hst1 [12]. Therefore, it may be deduced that endosomes might also be involved in the internalization and transition to mitochondria and ER. With this assumption, Hst1_12-22_ may lose its ability to escape endosomes, thus fusing with lysosomes. Further studies are highly needed to uncover the molecular mechanisms underlying the Hst-cell interactions.

## Figures and Tables

**Table 1:** Peptides Amino acid sequences of histatin 1 (Hst1 or Hst1_1-38_) and its variants without or with fluorescent label (F-).

Peptides	Amino Acid Sequences of Hst Variants
(F-) Hst1_1-38_	DSHEKRHHGYRRKFHEKHHSHREFPFYGDYGSNYLYDN
(F-) Hst2	RKFHEKHHSHREFPFYGDYGSNYLYDN
(F-) Hst5	DSHAKRHHGYKRKFHEKHHSHRGY
(F-) Hst1_1-11_	DSHEKRHHGYR
(F-) Hst1_12-22_	RKFHEKHHSHR
(F-) Hst1_23-38_	EFPFYGDYGSNYLYDN
(F-) Hst1^scr^	SDHSRHEEFKPRFHYHGGDYYRGRSKNFYHLEYKDHNH

Double underline indicates the amino acid linked to the fluorescent dye ATTO-647N.
